# Supplementary material for: T3SS translocon induces pyroptosis by direct interaction with NLRC4/NAIP inflammasome
Source: eLife. 2025 Feb 14;13:RP100820. doi: 10.7554/eLife.100820 (PMC11828483; doi:10.7554/eLife.100820)
Supplement: Supplementary file 1. [file elife-100820-supp1.docx]

**Supplementary File 1.** The primers used for the construction of *Edwardsiella tarda* translocon mutants.

| Primer | Sequence (5’-3’)^a^ | Mutant |
| --- | --- | --- |
| EseB/D-F1 | GGTTACCCGCATGCAGCAGCGCGCAGGCAAACGCG | Δ*eseB-D* |
| EseB/D R1 | AGAGCACTAGGAGGCACGATGGATGACG |  |
| EseB/D-F2 | TGCCTCCTAGTGCTCTCCTCTGAGGGAT |  |
| EseB/D-R2 | CCCTTCTAGATAGATCTGGAGACGCCGCTCAACGCCT |  |
| EscA-F1 | GGTTACCCGCATGCACTGCGCCTTAGCCTGATCCTC | Δ*escA* |
| EscA-R1 | CGCTGCATCGTCAGTTCACACCGGTGACC |  |
| EscA-F2 | AACTGACGATGCAGCGTAGCGAGATCGTC |  |
| EscA-R2 | CCCTTCTAGATAGATCTCCAGTTCATGACGCTATTCAC |  |
| EseB-F1 | GGTTACCCGCATGCAGTCTCCTCGGTCACCGGTGTG | Δ*eseB* |
| EseB-R1 | TAAACCACGCCGAGATGAACAAATCCATC |  |
| EseB-F2 | ATCTCGGCGTGGTTTACACCGCCGTGGTA |  |
| EseB-R2 | CCCTTCTAGATAGATCTGATAACCAAACAGCAAACGTT |  |
| EseC-F1 | GGTTACCCGCATGCACCGCTTAAGCTGCTGCTCGGC | Δ*eseC* |
| EseC-R1 | CTGAGACCGGCGCCATGCTCAGCAACATC |  |
| EseC-F2 | ATGGCGCCGGTCTCAGTGATATTGTTCAT |  |
| EseC-R2 | CCCTTCTAGATAGATCTAACAAATCCATCGCCCAGAAT |  |
| EseD-F1 | GGTTACCCGCATGCATTATCAGGGCGTGCGCCCCGG | Δ*eseD* |
| EseD-R1 | ACGGTGTTGCCCACGAACGTATCGCCAGC |  |
| EseD-F2 | TCGTGGGCAACACCGTGGCTGCCGCTGTC |  |
| EseD-R2 | CCCTTCTAGATAGATCTGCCGAGCAGAGCGGTCGCTTC |  |
| FliC-F1 | GGTTACCCGCATGCAGTATACAAATCAGTCACGTCG | Δ*fliC* |
| FliC-R1 | CGCTTCACCGTACAGAACCGTTTCGATTCC |  |
| FliC-F2 | GTTCTGTACGGTGAAGCGGTTGGAGATCG |  |
| FliC-R2 | CCCTTCTAGATAGATCTTACGCGTTATCGGCTCTGTTG |  |

^a^The underlined sequences are the homologous arm sequences in pDM4.
