# Supplementary material for: T3SS translocon induces pyroptosis by direct interaction with NLRC4/NAIP inflammasome
Source: eLife. 2025 Feb 14;13:RP100820. doi: 10.7554/eLife.100820 (PMC11828483; doi:10.7554/eLife.100820)
Supplement: Supplementary file 2. [file elife-100820-supp2.docx]

**Supplementary File 2.** Translocator proteins used in this study

| Protein | Bacteria | Accession number^*^ | Identity (%) with *Edwardsiella tarda* EseB |
| --- | --- | --- | --- |
| EspA_Av_ | *Aeromonas veronii* | QMS76431 | 77.3 |
| EspA_Pf_ | *Pseudogulbenkiania ferrooxidans* | ERE19475 | 76.8 |
| SseB_Bl_ | *Burkholderia lata* | VWD65036 | 64.2 |
| EspA_Sb_ | *Shewanella baltica* | AEH13802 | 51.0 |
| EspA_Pcu_ | *Parashewanella curva* | RLV59315 | 50.5 |
| SseB_Ae_ | *Arsenophonus endosymbiont* | A0A3B0MKK6  (UniProtKB accession number) | 50.2 |
| EspA_Vp_ | *Vibrio pectenicida* | NOH71600 | 48.8 |
| EspA_Eb_ | *Enterobacteriaceae bacterium* | QLK63763 | 37.7 |
| HypP_Rb_ | *Rouxiella badensis* | ORJ26452 | 36.5 |
| EspA_Pr_ | *Providencia rettgeri* | AVL73471 | 35.4 |
| EspA_Wi_ | *Winslowiella iniecta* | KOC86666 | 34.8 |
| EspA_Mc_ | *Mycoavidus cysteinexigens* | BBE08751 | 34.1 |
| EspA_Cv_ | *Chromobacterium vaccinii* | AVG16459 | 33.3 |
| HypP_Cv_ | *Chromobacterium violaceum* | AAQ60250 | 33.3 |
| SseB_Se_ | *Salmonella enterica* SPI-2 | HAF8569287 | 33.0 |
| HypP_Pc_ | *Pantoea cypripedii* | QGY32172 | 31.1 |
| SseB_Ha_ | *Hafnia alvei* | SCM50665 | 30.6 |
| EspA_Yr_ | *Yokenella regensburgei* | QIU89219 | 30.0 |
| HypP_Op_ | *Obesumbacterium proteus* | AMO83764 | 29.7 |
| EspA_Pch_ | *Pseudomonas chlororaphis* | QLL16699 | 28.5 |
| EspA_EPEC_ | enteropathogenic *Escherichia coli* | WP_000381567 | 22.8 |
| EspA_EHEC_ | enterohaemorrhagic *Escherichia coli* | WP_000381516 | 20.4 |
| EspA_Ea_ | *Escherichia albertii* | WP_000381555 | 20.4 |
| EspA_Cr_ | *Citrobacter rodentium* | AAL06381 | 18.8 |
| IpaC | *Shigella flexneri* | NP_858260 | 13.0 |
| IpaD | *Shigella flexneri* | ADA76864 | 12.9 |
| SipD | *Salmonella enterica* SPI-1 | NP_461804 | 12.8 |
| SipC | *Salmonella enterica* SPI-1 | NP_461805 | 10.4 |
| YopD | *Yersinia enterocolitica* | WP_010891207 | 9.7 |
| PcrV | *Pseudomonas aeruginosa* | NP_250397 | 9.6 |
| LcrV | *Yersinia enterocolitica* | WP_014609483 | 9.1 |
| PopD | *Pseudomonas aeruginosa* | NP_250400 | 8.0 |
| SseD | *Salmonella enterica* SPI-2 | AAC28882 | 7.4 |
| EspB | *enterohaemorrhagic Escherichia coli* | NP_312581 | 4.0 |

^*^, GenBank accession number unless otherwise indicated.

HypP, hypothetical or unnamed protein
